# The incidence and prevalence of inflammatory bowel disease in UK primary care: a retrospective cohort study of the IQVIA Medical Research Database

**DOI:** 10.1186/s12876-021-01716-6

**Published:** 2021-03-26

**Authors:** Karoline Freeman, Ronan Ryan, Nicholas Parsons, Sian Taylor-Phillips, Brian H. Willis, Aileen Clarke

**Affiliations:** 1 Warwick Medical School, University of Warwick, Coventry, UK; 2 Institute of Applied Health Research, University of Birmingham, Birmingham, UK

**Keywords:** Inflammatory bowel disease, Primary health care, Epidemiology, Electronic health care records

## Abstract

**Background:**

Our knowledge of the incidence and prevalence of inflammatory bowel disease (IBD) is uncertain. Recent studies reported an increase in prevalence. However, they excluded a high proportion of ambiguous cases from general practice. Estimates are needed to inform health care providers who plan the provision of services for IBD patients. We aimed to estimate the IBD incidence and prevalence in UK general practice.

**Methods:**

We undertook a retrospective cohort study of routine electronic health records from the IQVIA Medical Research Database covering 14 million patients. Adult patients from 2006 to 2016 were included. IBD was defined as an IBD related Read code or record of IBD specific medication. Annual incidence and 12-month period prevalence were calculated.

**Results:**

The prevalence of IBD increased between 2006 and 2016 from 106.2 (95% CI 105.2–107.3) to 142.1 (95% CI 140.7–143.5) IBD cases per 10,000 patients which is a 33.8% increase. Incidence varied across the years. The incidence across the full study period was 69.5 (95% CI 68.6–70.4) per 100,000 person years.

**Conclusions:**

In this large study we found higher estimates of IBD incidence and prevalence than previously reported. Estimates are highly dependent on definitions of disease and previously may have been underestimated.

**Supplementary Information:**

The online version contains supplementary material available at 10.1186/s12876-021-01716-6.

## Background

Inflammatory bowel disease (IBD) includes a group of related, chronic relapsing disorders. They place significant demand on healthcare resources including consultation time, testing and treatment. In order to plan healthcare resources, knowledge of the size of the problem is required. This can be inferred from the incidence and prevalence of IBD in the population. A recent systematic review published in the Lancet assessed the incidence and prevalence of IBD around the world [1]. Studies using UK data from the 1990s reported incidence rates ranging from 21 to 32.2/100,000 [2–4] and prevalence estimates ranging from 328 to 409/100,000 [2, 5–7]. The review suggested that incidence rates have stabilised in the western world, while other studies reported an ongoing increase in incidence rates [8, 9]. Two recent UK studies, that excluded a high proportion of ambiguous diagnoses from general practice, reported considerably higher prevalence estimates of 725–781/100,000 [10, 11]. A third recent study reported estimates for ulcerative colitis and Crohn’s disease but excluded cases of IBD unclassified (IBDU) [12]. However, IBD cannot be classified in 20–30% of patients at first presentation and 13% remain unclassified 1 year later [13]. This may have resulted in underestimates of the true IBD prevalence in UK general practice. Our aim is to establish estimates of incidence and prevalence of IBD in adult patients in UK general practice using routine primary care electronic health records.

UK primary care data, such as the IQVIA Medical Research Database (IMRD-UK) [formerly known as the Health Improvement Network (THIN)], are unique and particularly suitable for research. Over 95% of the UK population is registered with a GP [2, 14]. General practitioner (GPs) act as gatekeepers to all services and specialists in secondary care (excluding emergency care). Patients are usually only registered with one GP at any one point in time; and for each patient the registration date and the date when the patient leaves the practice is known. This provides longitudinal data with known start and end date of follow-up. The role of the GP extends to the management of chronic patients.

The IMRD population is broadly representative of the UK population and prevalence of chronic diseases is comparable to national rates [15]. Findings can be generalised to the broader UK primary care population [15].

## Methods

### Data source

Study data consisted of electronic health care records available in the IMRD. The IMRD consists of anonymised, longitudinal individual level patient data from more than 670 UK GP practices using the Vision practice software. In 2015 a total of over 14 million patients had contributed data to IMRD which reflects a coverage of about 6% of the UK population [16]. Data are based on patient consultation information including symptoms, diagnoses, investigations and medications recorded as clinical codes. Data were included into the study from GP practices from the date that the practice was deemed to be reporting all-cause mortality reliably compared to national statistics and from 1 year after the installation of the electronic medical record system. We applied these quality control measures to ensure data reliability and completeness.

The IMRD has received Research Ethics Committee approval by the NHS South-East Multicentre Ethics Committee for research as a whole. Scientific Review Committees (SRCs) have been established to review IMRD study protocols for scientific merit and feasibility. This project was given approval by the SRC (SRC Reference Number 17THIN089) on 23rd October 2017.

### Study design and study population

We undertook a retrospective cohort study of patients with data in the IMRD who were at least 18 years of age during the period 1 January 2006 to 31 December 2016. The study cohort was dynamic with patients entering and exiting the study at different times. Patients entered the study 1 year after they registered with the GP practice or at age 18 years, whichever came later. Patients exited the study at the earliest of the following dates: deregistration with the practice; death; or 1 January 2017.

### Definition of IBD diagnosis

The outcome of interest was newly diagnosed IBD. We searched the medical records of the study population for patients with a diagnosis of IBD. Those with a clinical code indicative of IBD and/or at least one prescription of an IBD specific medication in the patient record were classified as cases of IBD. The date of IBD diagnosis was taken as the first occurrence of a clinical code for IBD or first prescription of IBD specific medication in the patient record. We were interested in the broad category of inflammatory bowel disease and included clinical codes for general IBD, ulcerative colitis, Crohn’s disease, indeterminate colitis and microscopic colitis. Clinical code lists were adapted from those used in previous literature [6, 17]. IBD specific medication included mesalazine, olsalazine and balsalazide. Sulfasalazine, prednisolone and budesonide preparations were considered IBD specific if rectal. Preparations of beclometasone needed to clearly specify use for the bowel to be included. Therefore, the definitions for medications were purposefully narrow and decisions on inclusion were exclusive if in doubt. The complete code list to identify IBD diagnoses is available in Additional file 1.

### Analysis

The annual incidence and 12-month period prevalence of IBD were determined for 2006–2016 considering all adult patients contributing data to IMRD in that period. Annual incidence was defined as the number of new cases of IBD during a 1 year period over the total time each patient was observed (person-time at risk). Period prevalence was defined as new and pre-existing IBD cases during a 12-month period over the number of patients in the IMRD database during the same time period. Confidence intervals for incidence rates were exact Poisson confidence limits. Confidence intervals for prevalence were calculated using the Wilson procedure for proportions without a correction for continuity. Incidence rates for male and female patients were compared using the two sample z test.

All analyses were undertaken in R version 3.6.1 (Vienna, Austria) [18]. The package “epitools” was used to calculate exact confidence intervals for incidence rates [19]. Graphs were drawn using the package “ggplot2” [20].

## Results

### IBD incidence and prevalence

**Fig. 1:**
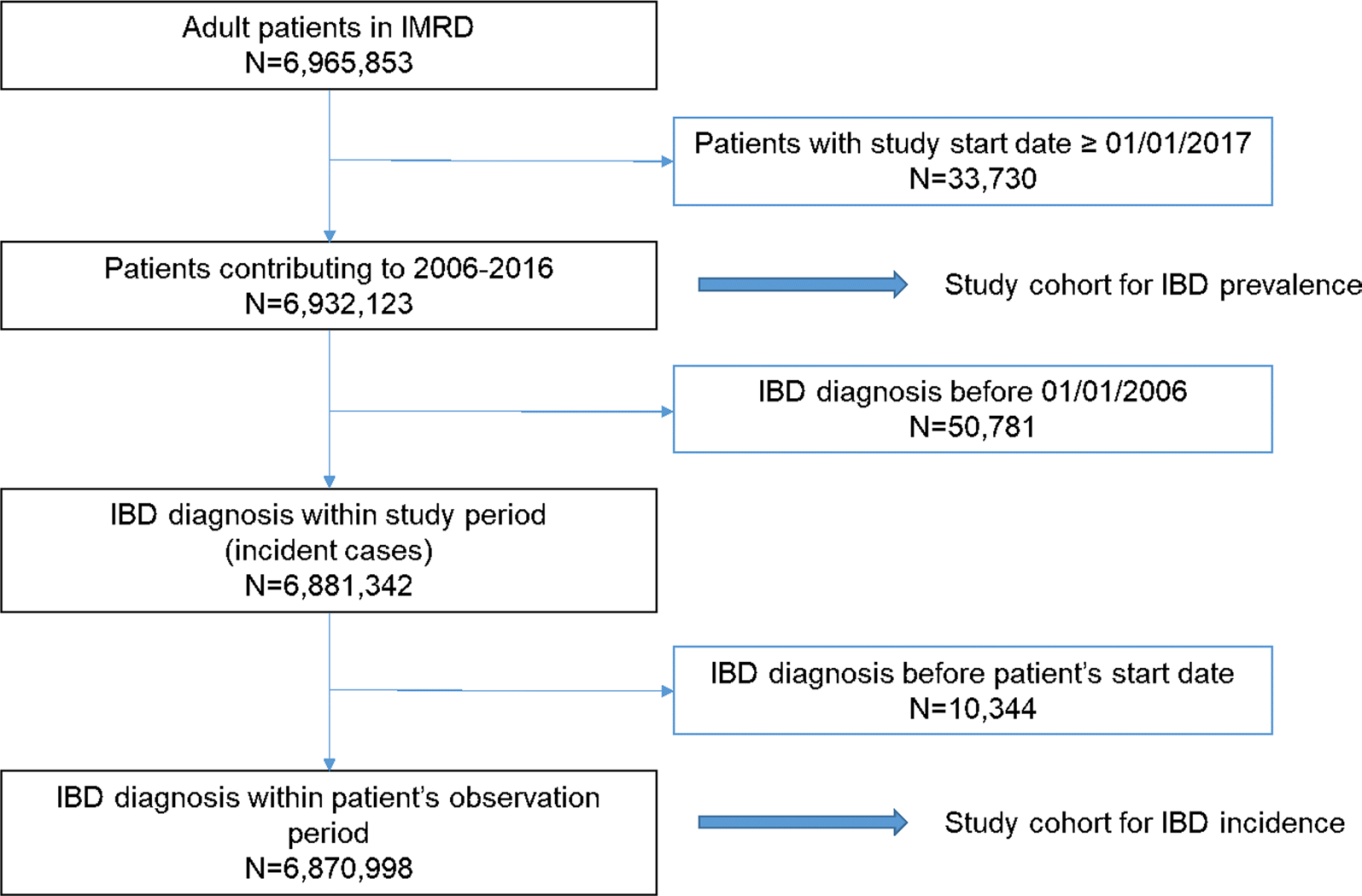
Overview of inclusion and exclusion of cases for the analyses of IBD incidence and prevalence

**Fig. 2:**
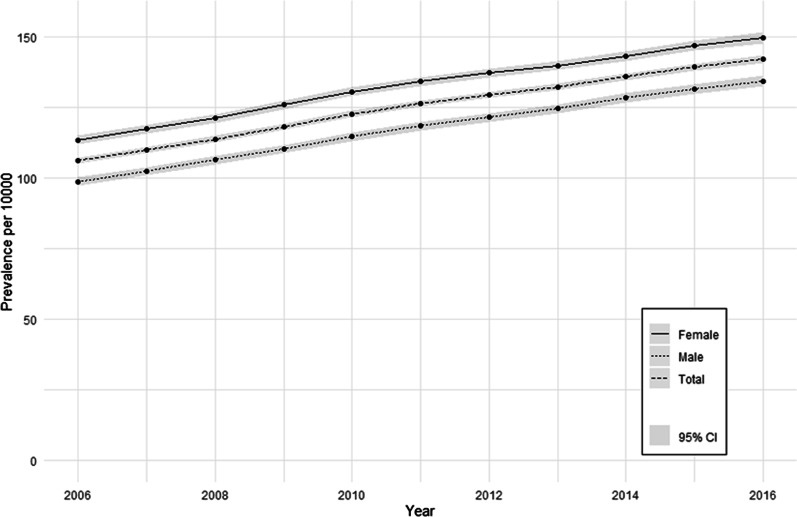
12-month period prevalence of IBD per 10,000 adult IMRD population

We retrieved 6,965,853 records of adult patients from the IMRD database and excluded 33,730 patients who entered the study after the study period (Fig. 1). We included a total of 6,932,123 patients in the analysis of IBD prevalence. The prevalence of IBD increased between 2006 and 2016 from 106.2 (95% CI 105.2–107.3) to 142.1 (95% CI 140.7–143.5) IBD cases per 10,000 in the adult IMRD population with an average increase of 2.96% per annum. This amounts to an increase of 33.8% from 2006 to 2016. More women than men had a recorded diagnosis of IBD (Fig. 2).

**Fig. 3:**
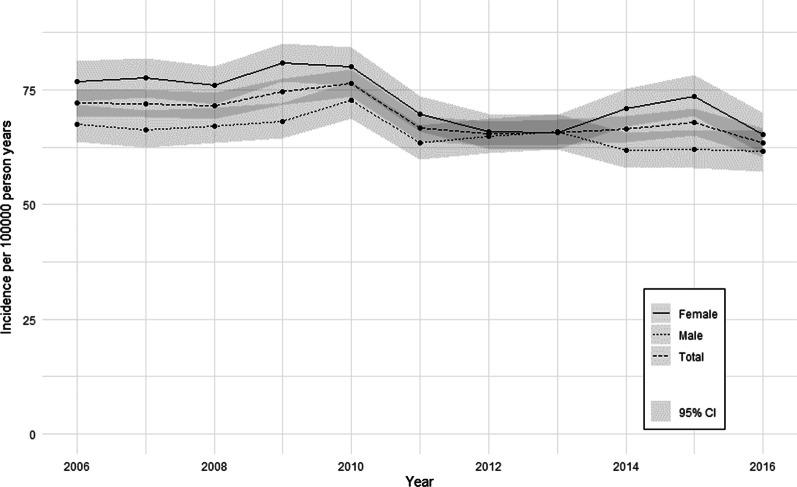
Annual IBD incidence per 100,000 person-years in the adult IMRD population

We excluded 61,125 prevalent IBD cases from the dataset which resulted in a dataset of 6,870,998 patients for the analysis of IBD incidence (Fig. 1). There were 25,470 IBD incidence cases between 2006 and 2016. 4736 (18.6%) had an IBD Read code only, 9632 (37.8%) had a prescription of an IBD medication only and 11,102 (43.6%) had both. Incidence of IBD in the adult IMRD population varied across the years with a maximum of 76.4 (95% CI 73.6–79.4) per 100,000 recorded in 2010 and the lowest incidence of 63.5 (95% CI 60.4–66.7) per 100,000 recorded in 2016 (Fig. 3). The incidence across the full study period was 69.5 (95% CI 68.6–70.4) per 100,000 person years. The incidence rate was higher in women than men for the study period (73.09 versus 65.83, z = 8.3, *p* < 0.0001).

## Discussion

### Summary of study findings

The analyses of IBD prevalence and incidence included a total of 6,932,123 and 6,870,998 adult patients, respectively. The prevalence of IBD in 2016 was 142.1 (95% CI 140.7–143.5) per 10,000 adult patients. The prevalence of IBD increased between 2006 and 2016 by 33.8%. This is likely due to the fact that IBD is a chronic condition which is associated with a low mortality rate. The mean IBD incidence for the study period was 69.3 (95% CI 66.8–71.8) per 100,000 person years. The drop in incidence between 2010 and 2011 may be an artefact or caused by an administrative change in coding/reporting standards. Over the most recent 5-year period, the incidence of IBD was relatively stable.

### Study strengths and limitations

The IQVIA Medical Research database is a rich source of routine electronic health care records of patients managed in primary care and is particularly useful for the study of real world problems. The study population was large and covered nearly 50% of all UK Clinical Commissioning Groups [16] meaning that findings are generalisable to UK primary care in general.

The criterion “registration date plus 1 year” to assess patients’ eligibility for study inclusion avoided the systematic over-reporting of incidence rates in the first year of follow-up for newly registered patients [21]. It also prevented the double counting of prevalent cases when patients transfer from one IMRD practice to another.

Limitations that might have affected the research are linked to characteristics of routine data.

IBD diagnoses might be missing either due to incorrect coding, missed coding or recording as free-text. This might have led to an underestimation of IBD incidence and prevalence. However, we included a record of an IBD specific medication in the definition of an IBD diagnosis which mitigated the effect. This may explain our higher figures for IBD incidence and prevalence when compared to a recent study which only included patients with two IBD Read codes recorded or one IBD Read code and an IBD drug code [11].

Potential misclassification through miscoding of ulcerative colitis as Crohn’s disease and vice versa, or by using higher order codes rather than disease specific codes was of no consequence to our study. We were interested in the broad category of inflammatory bowel disease rather than sub-category, severity or location of disease. We were able to include codes for IBD and indeterminate IBD and present the complete picture of IBD in primary care which is in contrast to a recent study which only focused on patients with a diagnosis of ulcerative colitis or Crohn’s disease [12].

A limitation of our study may be our inability to verify IBD cases. While we mitigated against under-coding, over-coding is a possibility. A study reported that about 6% of IBD codes did probably not relate to a true IBD diagnosis [6]. However, the study relied on confirmatory data from GP questionnaires and considered coded data from 20 years ago.

### Findings in the context of existing literature

Published figures on UK IBD incidence rates range from 21 to 37.5/100,000 [2–4, 10–12]. Studies consistently report that prevalence is rising worldwide because of the low mortality associated with this chronic condition. UK prevalence estimates range from 328/100,000 in the 1990s [6] to 970/100,000 in 2017 [12].

Our estimates of incidence and prevalence of IBD in the UK are about 1.8 and 1.5 times higher than the most recent estimates. Published studies are very heterogeneous, complicating comparison of reported rates across studies. Major variations that explain at least some of the differences include: (1) our study included adult patients only, while the majority of other studies covered a wider age range including children. This impacts the incidence and prevalence rates of IBD which has an onset that peaks in adulthood. (2) Improvements in diagnostic technology now enable the detection of milder cases [9]. (3) Some smaller studies used GP records to identify cases with subsequent exclusion of unverified cases. Exclusions ranged from 8 to 26% of patients [2, 3, 10]. This could have underestimated true IBD prevalence. (4) Studies used different definitions of disease. A number of studies did not include indeterminate IBD or microscopic IBD in their definition. A recent study reported the incidence and prevalence of ulcerative colitis and Crohn’s disease in the IMRD-UK database [12]. The study only included Read codes for Crohn’s disease and ulcerative colitis in the definition of disease. In contrast we used a very comprehensive and sensitive list of Read codes and drug codes (48 codes) for the identification of IBD, ulcerative colitis, Crohn’s disease, indeterminate IBD and microscopic colitis. In addition, a previous study using the IMRD-UK data used a similar list of Read codes to our study for the identification of IBD. However, they included non-specific IBD medications to identify IBD cases and only included patients with at least two subsequent IBD records or an IBD record and a recorded prescription of an IBD related drug [11]. According to our data, this approach may have missed at least 37.8% of cases. We were able to increase the sensitivity of our Read code list by using medications to identify additional IBD cases because we restricted inclusion of prescriptions to IBD specific medications. This is an advantage of our study over these two recent IMRD-UK studies.

### Implications for research and practice

Taken together, the evidence suggests that the IBD incidence and prevalence in the UK adult population may be higher than the latest published figures. Some of the differences in reported rates may be due to differences in methodology including differences in methods of case definition [22]. Case definition is complicated by the fact that IBD is a heterogeneous group of disorders. Crohn’s disease and ulcerative colitis are considered as the two extremes of a spectrum of chronic gut disorders [23]. Furthermore, the phenotype of IBD is not uniform resulting in IBD unclassified cases [13, 24]. The overlap with other infectious, inflammatory and autoimmune disorders led to suggestions to diverge from the classification of IBD into ulcerative colitis and Crohn’s disease and to reclassify IBD considering a broader disease spectrum [25]. This argues for a broader definition of IBD in the estimation of IBD incidence and prevalence.

## Conclusions

In this large study we found higher estimates of IBD incidence and prevalence than previously reported. Estimates are highly dependent on definitions of disease and previously may have been underestimated. We believe that our sensitive approach to identifying IBD cases may be more reflective of the true burden of disease in UK general practice. Health care providers who plan services for IBD patients need to make allowances for these updated figures and should consider the definition of disease in published studies.

## Supplementary Information

**Additional file 1**. Complete list of clinical and drug codes for the identification of IBD diagnoses in electronic health records.

## Data Availability

The datasets generated during and/or analysed during the current study are not publicly available under the data sharing agreement with the University of Birmingham on behalf of IQVIA.

## Abbreviations

IBD: Inflammatory bowel disease
IMRD: IQVIA Medical Research Database
THIN: The health improvement network
GP: General practitioner
CI: Confidence interval
IBDU: IBD unclassified
