# Supplementary material for: The incidence and prevalence of inflammatory bowel disease in UK primary care: a retrospective cohort study of the IQVIA Medical Research Database
Source: BMC Gastroenterol. 2021 Mar 26;21:139. doi: 10.1186/s12876-021-01716-6 (PMC8004426; doi:10.1186/s12876-021-01716-6)
Supplement: Supplementary file 1 — Additional file 1. Complete list of clinical and drug codes for the identification of IBD diagnoses in electronic health records. [file 12876_2021_1716_MOESM1_ESM.docx]

**Additional Files**

Table S1 IBD Read code list

| **Variable** | **medcode** | **Description** |
| --- | --- | --- |
| IBD | 14C4.11 | H/O: ulcerative colitis |
|  | J08z900 | Orofacial Crohn's disease |
|  | J4...12 | Inflammatory bowel disease |
|  | J40..00 | Regional enteritis - Crohn's disease |
|  | J40..11 | Crohn's disease |
|  | J40..12 | Granulomatous enteritis |
|  | J400.00 | Regional enteritis of the small bowel |
|  | J400000 | Regional enteritis of the duodenum |
|  | J400100 | Regional enteritis of the jejunum |
|  | J400200 | Crohn's disease of the terminal ileum |
|  | J400300 | Crohn's disease of the ileum unspecified |
|  | J400400 | Crohn's disease of the ileum NOS |
|  | J400500 | Exacerbation of Crohn's disease of small intestine |
|  | J400z00 | Crohn's disease of the small bowel NOS |
|  | J401.00 | Regional enteritis of the large bowel |
|  | J401000 | Regional enteritis of the colon |
|  | J401100 | Regional enteritis of the rectum |
|  | J401200 | Exacerbation of Crohn's disease of large intestine |
|  | J401z00 | Crohn's disease of the large bowel NOS |
|  | J401z11 | Crohn's colitis |
|  | J402.00 | Regional ileocolitis |
|  | J40z.11 | Crohn's disease NOS |
|  | J41..12 | Ulcerative colitis and/or proctitis |
|  | J410.00 | Ulcerative proctocolitis |
|  | J410000 | Ulcerative ileocolitis |
|  | J410100 | Ulcerative colitis |
|  | J410200 | Ulcerative rectosigmoiditis |
|  | J410300 | Ulcerative proctitis |
|  | J410400 | Exacerbation of ulcerative colitis |
|  | J410z00 | Ulcerative proctocolitis NOS |
|  | J411.00 | Ulcerative (chronic) enterocolitis |
|  | J412.00 | Ulcerative (chronic) ileocolitis |
|  | J413.00 | Ulcerative pancolitis |
|  | J436.00 | Microscopic colitis |
|  | J436000 | Collagenous colitis |
|  | J436100 | Lymphocytic colitis |
|  | J438.00 | Left sided colitis |
|  | J4z3.00 | Non-infective colitis NOS |
|  | J4z4.00 | Non-infective sigmoiditis NOS |
|  | J4z6.00 | Indeterminate colitis |
|  | Jyu4000 | [X]Other Crohn's disease |
|  | Jyu4100 | [X]Other ulcerative colitis |
|  | N031000 | Arthropathy in ulcerative colitis |
|  | N031100 | Arthropathy in Crohn's disease |
|  | N045300 | Juvenile arthritis in Crohn's disease |
|  | N045400 | Juvenile arthritis in ulcerative colitis |
|  | ZR3S.00 | Crohn's disease activity index |
|  | ZR3S.11 | CDAI - Crohn's disease activity index |

Table S2 Drug code list for IBD

| Mesalazine | 53913979 | Mesalazine 800mg gastro-resistant tablets |
| --- | --- | --- |
|  | 88517998 | Mesalazine 400mg gastro-resistant tablets |
|  | 93624997 | Mesalazine 250mg modified release tablets |
|  | 99583998 | Mesalazine 250mg modified-release tablet |
|  | 54552979 | Mesalazine 400mg gastro-resistant tablets |
|  | 55164978 | Mesalazine 4g modified-release granules sachets sugar free |
|  | 55165978 | Mesalazine 4g modified-release granules sachets sugar free |
|  | 58800979 | Mesalazine 1.2g gastro-resistant modified-release tablets |
|  | 60584979 | Mesalazine 3g gastro-resistant modified-release granules sachets sugar free |
|  | 60585979 | Mesalazine 3g gastro-resistant modified-release granules sachets sugar free |
|  | 64868979 | Mesalazine 2g modified-release granules sachets sugar free |
|  | 76424978 | Mesalazine 1g modified-release tablets |
|  | 79867978 | Mesalazine 400mg gastro-resistant tablets |
|  | 80928998 | Mesalazine 3g gastro-resistant modified-release granules sachets sugar free |
|  | 80929998 | Mesalazine 3g gastro-resistant modified-release granules sachets sugar free |
|  | 81193998 | Mesalazine 1g modified-release tablets |
|  | 81194998 | Mesalazine 1g modified-release tablets |
|  | 81683998 | Mesalazine 1g suppositories |
|  | 81689998 | Mesalazine 500mg gastro-resistant tablets |
|  | 81690998 | Mesalazine 500mg gastro-resistant tablets |
|  | 81772998 | Mesalazine 800mg gastro-resistant tablets |
|  | 81868998 | Mesalazine 400mg gastro-resistant tablets |
|  | 83503998 | Mesalazine 1.5g gastro-resistant modified-release granules sachets sugar free |
|  | 83504998 | Mesalazine 1.5g gastro-resistant modified-release granules sachets sugar free |
|  | 83743998 | Mesalazine 2g modified-release granules sachets sugar free |
|  | 83987998 | Mesalazine 2g modified-release granules sachets sugar free |
|  | 84059998 | Mesalazine 1g gastro-resistant modified-release granules sachets sugar free |
|  | 84209998 | Mesalazine 800mg gastro-resistant tablets |
|  | 84290998 | Mesalazine 1.2g gastro-resistant modified-release tablets |
|  | 84291998 | Mesalazine 1.2g gastro-resistant modified-release tablets |
|  | 85560998 | Mesalazine 800mg gastro-resistant tablets |
|  | 87761998 | Mesalazine 400mg gastro-resistant tablets |
|  | 87909998 | Mesalazine 1g gastro-resistant modified-release granules sachets sugar free |
|  | 87910998 | Mesalazine 500mg gastro-resistant modified-release granules sachets sugar free |
|  | 87911998 | Mesalazine 500mg gastro-resistant modified-release granules sachets sugar free |
|  | 89992997 | Mesalazine 2g/59ml enema |
|  | 89992998 | Mesalazine 500mg suppositories |
|  | 92346998 | Mesalazine 1g/application foam enema |
|  | 92347998 | Mesalazine 400mg gastro-resistant tablets |
|  | 92764997 | Mesalazine 1g modified-release granules sachets sugar free |
|  | 92764998 | Mesalazine 500mg modified-release tablets |
|  | 93623996 | Mesalazine 250mg suppositories |
|  | 93623997 | Mesalazine 1g/application foam enema |
|  | 93623998 | Mesalazine 1g/100ml enema |
|  | 93624996 | Mesalazine 1g suppositories |
|  | 93624998 | Mesalazine 1g/100ml enema |
|  | 93728992 | Mesalazine 500mg modified-release tablets |
|  | 94564992 | Mesalazine 500mg modified-release tablets |
|  | 95041990 | Mesalazine 400mg gastro-resistant tablets |
|  | 95888997 | Mesalazine 250mg gastro-resistant tablets |
|  | 95888998 | Mesalazine 400mg gastro-resistant tablets |
|  | 96608996 | Mesalazine 2g/59ml enema |
|  | 96608997 | Mesalazine 1g suppositories |
|  | 96608998 | Mesalazine 500mg suppositories |
|  | 96659996 | Mesalazine 1g/application foam enema |
|  | 96659997 | Mesalazine 500mg suppositories |
|  | 96659998 | Mesalazine 250mg suppositories |
|  | 96883990 | Mesalazine 400mg gastro-resistant tablets |
|  | 96916992 | Mesalazine 500mg modified-release tablets |
|  | 97381998 | Mesalazine 400mg gastro-resistant tablets |
|  | 97764998 | Mesalazine 250mg gastro-resistant tablets |
|  | 98001992 | Mesalazine 250mg gastro-resistant tablets |
|  | 99486979 | Mesalazine 400mg gastro-resistant tablets |
|  | 99487979 | Mesalazine 400mg gastro-resistant tablets |
|  | 99488979 | Mesalazine 400mg gastro-resistant tablets |
|  | 99490979 | Mesalazine 1g modified-release granules sachets sugar free |
|  | 99492979 | Mesalazine 1g suppositories |
|  | 99494979 | Mesalazine 1g suppositories |
|  | 99495979 | Mesalazine 500mg modified-release tablets |
|  | 99498979 | Mesalazine 500mg modified-release tablets |
|  | 99583996 | Mesalazine 1g gastro-resistant modified-release granules sachets sugar free |
|  | 99583997 | Mesalazine 500mg modified-release tablets |
| Olsalazine | 92400998 | Olsalazine 500mg tablets |
|  | 92401998 | Olsalazine 250mg capsules |
|  | 94437997 | Olsalazine 500mg tablets |
|  | 94437998 | Olsalazine 250mg capsules |
|  | 94438997 | Olsalazine 500mg tablets |
|  | 94438998 | Olsalazine 250mg capsules |
| Balsalazide | 88489998 | Balsalazide 750mg capsules |
|  | 88492998 | Balsalazide 750mg capsules |
| Sulfasalazine | 89598997 | Sulfasalazine 3g/100ml retention enema |
|  | 89598998 | Sulfasalazine 500mg suppositories |
|  | 89604997 | Sulfasalazine 3g/100ml retention enema |
|  | 89604998 | Sulfasalazine 500mg suppositories |
|  | 95256996 | Sulfasalazine 3g/100ml enema |
|  | 95256997 | Sulfasalazine 500mg suppositories |
|  | 97281996 | Sulfasalazine 3g/100ml retention enema |
|  | 97281997 | Sulfasalazine 500mg suppositories |
| Beclometasone | 86941998 | Beclometasone 5mg gastro-resistant modified-release tablets |
|  | 86942998 | Beclometasone 5mg gastro-resistant modified-release tablets |
| Budenoside | 50988978 | Budesonide 9mg modified-release tablets |
|  | 50989978 | Budesonide 9mg modified-release tablets |
|  | 60586979 | Budesonide 9mg gastro-resistant granules sachets |
|  | 60587979 | Budesonide 9mg gastro-resistant granules sachets |
|  | 80931998 | Budesonide 9mg gastro-resistant granules sachets |
|  | 80932998 | Budesonide 9mg gastro-resistant granules sachets |
|  | 84636998 | Budesonide 2mg foam enema |
|  | 84637998 | Budesonide 2mg foam enema |
|  | 89238998 | Budesonide 2mg/100ml enema |
|  | 89239998 | Budesonide 2mg/100ml enema |
|  | 94125992 | Cortenema enema ml liq |
| Prednisolone | 60097979 | Prednisolone 20mg/application foam enema |
|  | 84741998 | Prednisolone 40mg/100ml enema |
|  | 90310979 | Prednisolone 20mg/application foam enema |
|  | 93706998 | Prednisolone sodium phosphate 5mg suppositories |
|  | 94336997 | Prednisolone sodium phosphate 5mg suppositories |
|  | 94336998 | Prednisolone rectal ointment |
|  | 94451998 | Prednisolone 20mg/application foam enema |
|  | 94452998 | Prednisolone 20mg/application foam enema |
|  | 94468998 | Prednisolone 20mg/100ml enema standard tube |
|  | 98370998 | Prednisolone sodium phosphate 5mg suppositories |
|  | 98371998 | Prednisolone 20mg/100ml enema standard tube |
|  | 99227997 | Prednisolone 20mg/100ml enema standard tube |
|  | 99227998 | Prednisolone 20mg/100ml enema standard tube |
|  | 99388979 | Prednisolone 20mg/100ml enema standard tube |
|  | 99389979 | Prednisolone 20mg/100ml enema standard tube |
|  | 89284997 | Cinchocaine 1mg / Prednisolone hexanoate 1.3mg suppositories |
